# Aberrant Pyramidal Tract in Comparison with Pyramidal Tract on Diffusion Tensor Tractography: A Mini-Review

**DOI:** 10.3389/fneur.2017.00314

**Published:** 2017-06-28

**Authors:** Sungho Jang, Soyoung Kwak

**Affiliations:** ^1^Department of Physical Medicine and Rehabilitation, College of Medicine, Yeungnam University, Daegu, South Korea

**Keywords:** diffusion tensor tractography, diffusion tensor imaging, pyramidal tract, aberrant pyramidal tract, stroke, brain injury

## Abstract

The pyramidal tract (PT) is a major neural tract that controls voluntary movements in the human brain. The PT has several collateral pathways, including the aberrant pyramidal tract (APT), which passes through the medial lemniscus location at the midbrain and pons. Diffusion tensor tractography (DTT) allows visualization and estimation of the APT in three dimensions. In this mini-review, eight DTT studies on the APT were reviewed. Two studies for normal subjects reported the prevalence (17–18% of hemispheres) and the different characteristics (different cortical origin, less directionality, and fewer neural fibers) of the APT compared with the PT. Six studies reported on the APT in patients with cerebral infarct, traumatic brain injury, and cerebral palsy and suggested that the APT could contribute to motor recovery following brain injury. The research on the APT in patients with brain injury has important implications for neuro-rehabilitation because understanding of the motor recovery mechanism can provide the basis for scientific rehabilitation strategies. Therefore, studies involving various brain pathologies with large numbers of patients on this topic should be encouraged. In addition, further studies are needed on the exact role of the APT in normal subjects.

## Introduction

The pyramidal tract (PT) is a major neural tract that controls voluntary movements in the human brain (1). The PT descends through the posterior corona radiata, the posterior limb of the internal capsule, the cerebral peduncle of the midbrain, the anterior pons, and the anterior medulla. The PT has several collateral pathways, including the aberrant pyramidal tract (APT), which passes through the medial lemniscus location at the midbrain and pons (2–6). The APT has been described using autopsy, electrophysiological, and radiological methods (5, 7–10). However, these methods are limited in that they cannot visualize the APT or be applied in the live human brain.

Diffusion tensor tractography (DTT), derived from diffusion tensor imaging (DTI), introduced in 1990s, allows visualization and estimation of the PT in three dimensions (11, 12). A few studies using DTT have reconstructed the APT and described the characteristics of the APT in the normal human brain (13, 14). Several studies using DTT reported that the APT plays a critical role in recovery mechanisms of motor function in patients with brain injury (15–20).

In this mini-review, DTT studies of the APT were reviewed. Relevant studies were identified using the electronic databases Pubmed and MEDLINE, from 1966 to 2017. The following key words were used: DTI, DTT, PT, APT, corticospinal tract, stroke, traumatic brain injury, motor recovery, and brain plasticity. This review was limited to studies of humans. Finally, eight studies were selected and reviewed (13–20) (Table 1).

**Table 1:** Diffusion tensor tractography studies of the aberrant pyramidal tract.

Authors	Publication year	Number of subjects	Pathology	Location of lesion	Timing of DTI after onset	Other Combined evaluation
**Normal subjects**						
Hong et al. (13)	2009	14				
Kwon et al. (14)	2011	93				
**Patients with brain injury**						
Jang (15)	2009	1	Infarct	Pons	6 months	fMRI
Lindenberg (16)	2010	35	Infarct	MCA territory	5 months	
Yeo and Jang (17)	2011	1	Infarct	Cerebral peduncle	3 weeks, 20 months	TMS
Hong and Jang (18)	2011	1	Infarct	Corona radiata	6 months	fMRI
Meoded et al. (19)	2012	1	Corticospinal tract malformation	Cerebral peduncle, pons	3 years	
Yeo and Jang (20)	2013	1	Traumatic intracerebral hemorrhage	Corona radiata	13 months	TMS

*DTI, diffusion tensor imaging; fMRI, functional magnetic resonance imaging; TMS, transcranial magnetic stimulation; MCA, middle cerebral artery*.

## DTT Studies on the APT

### Studies on Normal Subjects

For normal subjects, two studies have reported on the APT using DTT (13, 14). In 2009, Hong et al. reported the prevalence and pathway of the APT in 14 normal subjects (13). They found that the APT was identified in 5 (17.9%) of the 28 hemispheres of normal subjects, and it descended through the medial lemniscus at the midbrain and pons level, and then rejoined the PT at the upper medulla.

**Figure 1:**
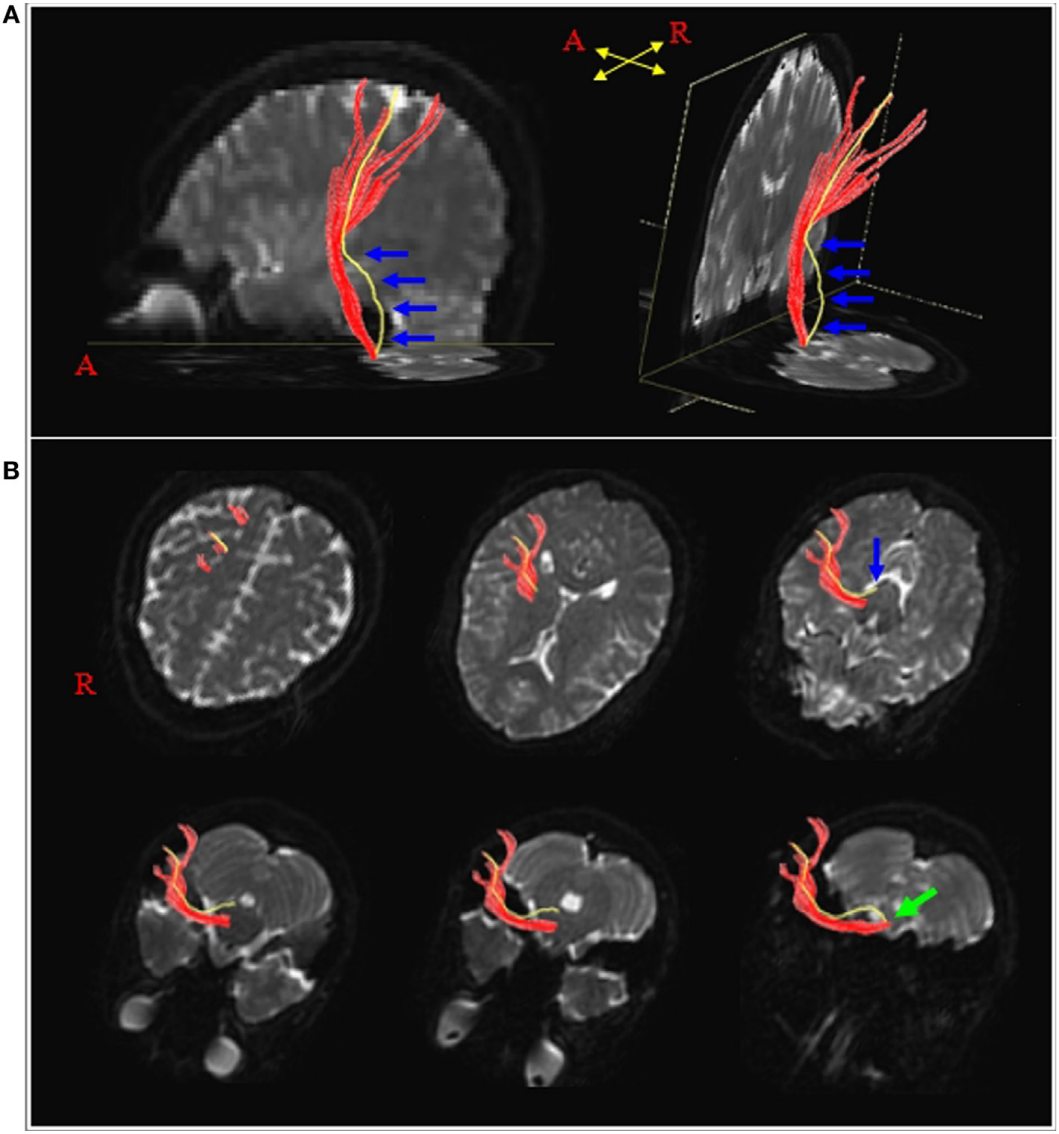
Results of diffusion tensor tractography for the pyramidal tract (PT) and the aberrant pyramidal tract (APT). **(A)** The PT and APT were constructed in the right hemisphere (red: the PT, yellow: the APT). The APT descended along the known pathway of the PT to the posterior limb of the internal capsule, then descended through the medial lemniscus from the midbrain to the pons (blue arrows). **(B)** The pathways of the PT and the APT are shown at the axial views (blue arrow: the APT was separated from the PT at the upper midbrain level, green arrow: the APT rejoined with the PT at the upper medulla level). Reprinted from “Characteristics of the APT in comparison with the PT in the human brain,” by Kwon et al. (14) with permission.

In 2011, Kwon et al. described the several characteristics of the APT in comparison with the PT in 93 normal subjects (14); first, the PTs always originated from the primary sensorimotor cortex (SM1) while 26.5% of the APTs originated from the primary somatosensory cortex without a primary motor cortex origin (Figure 1); second, the value of fractional anisotropy (FA) of the APT (0.53), which represents the degree of diffusion process along axonal bundles, accounting for white matter microstructural integrity (e.g., axons, myelin, and microtubules) ranging from 0 to 1, was lower than that of the PT (0.59) (21). Third, the tract volume, which indicates the number of voxels contained within the neural tract of the APT (191.65) was less than that of the PT (592.91) (22). The authors concluded that the APT can be described as having less origin from the primary motor cortex, less directionality, and fewer neural fibers than the PT.

### Studies on Patients with Brain Injury

To our best knowledge, six studies have reported on the APT in the patients with brain injury, including cerebral infarct, traumatic brain injury, and cerebral palsy (15–20). These studies suggest that the APT could contribute to motor recovery following brain injury.

In 2009, Jang reported on a patient whose motor function appeared to have recovered *via* an APT following a pontine infarct located in the PT area (15). The patient who had severe right hemiparesis due to an infarct in the left anterior pons slowly recovered to a nearly normal state over a period of 6 months after the onset. An APT, descending through the medial lemniscus from the midbrain to the pons, was detected in the left (affected) cerebral hemisphere on 6-month DTT. However, on the 6-month functional MRI, the contralateral primary SM1 was activated during the movements of affected hand as well as unaffected hand.

Lindenberg et al. (16) demonstrated that the patients with alternate motor fibers in the brainstem showed better motor function after middle cerebral artery infarcts (16). They recruited 35 chronic stroke patients with varying degrees of hemiparesis who underwent DTT at a chronic stage (at least 5 months from the onset). Fibers originating from the precentral gyrus were identified and separated into the PT and alternate motor fibers. Asymmetry indices of the values of FA and fiber number, comparing the affected to the unaffected hemispheres, were associated with the motor impairment scores. When all motor tracts (both the PT and alternate motor fibers) were taken into account, the asymmetry indices showed stronger correlation with the motor impairment scores than that of when only the PT was taken into account. The authors concluded that the integrity of all descending motor tracts including the alternate motor fibers, not merely the PT, appears to account for motor recovery following stroke. The alternate motor fibers appear to be the APT on their configurations although the authors did not specify that the alternate motor fibers were APTs.

In 2011, Yeo and Jang reported a patient with a cerebral infarct, who showed an APT on DTT (17). The patient presented with severe right hemiparesis due to an infarct in the left mid to lateral portion of the cerebral peduncle. The patient showed progressive motor recovery to the point of being able to extend the right extremities against some resistance at 6 months after onset. DTT was performed twice: at 3-week and 20-month from the onset. On both DTTs, the left PT was discontinued below the left infarction lesion in the midbrain; while the APT that descends through the medial lemniscus pathway from the midbrain to the pons was observed in the left hemisphere. In the transcranial magnetic stimulation performed 3 weeks after the onset, no motor evoked potential was evoked from the right (affected) hand muscle; however, a mildly delayed latency with low amplitude potential (compared to that of the left hand muscle) was evoked from the same hand muscle after 20 months from the onset (23).

During the same year, Hong and Jang (18) reported on a patient with severe right hemiparesis due to an infarct in the left corona radiata (18). The patient had recovered his motor function to the point that he can perform some fine motor activities, as well as walk with a nearly normal gait pattern at 6 months after the onset. On 6-month functional MRI, the contralateral primary SM1 was activated during movement of right hand as well as left hand. On 2-week DTT, an APT that bypassed through the medial lemniscus from the midbrain to the lower pons was observed along with the PT in the left hemisphere. However, on 6-month DTT, the PT from midbrain to pons in the left hemisphere was not reconstructed; instead, only the APT was identified along the course of the disappeared PT. The authors concluded that the results suggest that the main motor function of the right extremities appeared to be controlled by the APT with degeneration of the PT in the brainstem of the left (affected) hemisphere.

Meoded et al. evaluated a pediatric patient with mild left hemiparesis since birth (19). Conventional MR imaging at 3 years showed hemiatrophy of the right cerebral peduncles and pons. DTT at 3 years showed that the right PT was present but had a completely aberrant course (the medial lemniscus location) at the level of the pons, with thinning at the posterior limb level without presence of the PT.

Yeo and Jang (20) reported on a patient who had revealed right hemiparesis due to a traumatic intracerebral hemorrhage in the left corona radiata (20). The motor function of the patient recovered almost to the normal at 10 months after onset. On 13-month DTT, the left PT showed discontinuation at the pontine level. Instead, an APT originating from the primary motor cortex and the supplementary motor area, descending through the medial lemniscus pathway from the midbrain to the pons, and finally entering the PT area at the pontomedullary junction was identified. On 13-month transcranial magnetic stimulation, a motor-evoked potential with mildly delayed latency and low amplitude in the right (affected) hand muscle compared with that of the left hand muscle was observed (23). This suggested that the motor function of the right extremities in this patient was recovered by the APT.

## Conclusion

In this article, eight studies (two studies: normal subjects and six studies: brain injury) were reviewed. Among the normal subjects, the APT had different characteristics (different cortical origin, less directionality, and fewer neural fibers) compared with the PT. These results appeared to coincide with the results (mildly delayed latency and low amplitude) of motor-evoked potential for the APT in patients with brain injury (17, 20). Regarding the studies for patients with brain injury, the results suggest that the APT could contribute to motor recovery following brain injury. Elucidating the exact role of the APT both in normal subjects and patients with brain injury has important implications for neuro-rehabilitation, because the understanding of the motor recovery mechanism can provide the basis for scientific rehabilitation strategies. However, five out of six studies on patients with brain injury in our review were case reports and this might lead to bias in interpretation of the role of the APT following brain injury.

Therefore, future research would be needed to ascertain the exact role of APT in normal subjects. Also, more research involving various brain pathologies with large number of patients should be carried out to decide the exact clinical significance of the APT after brain injury. If the APT is associated with better recovery after brain injury, strategies to facilitate the APT should be identified as well. Finally, the limitation of DTT need to be addressed in the future research. Although DTT is a powerful anatomic imaging tool that can demonstrate the gross fiber architecture, the small neural tract such as the APT can be affected by partial volume effect (24).

## Author Contributions

SJ: conceiving and designing the study, funding, data acquisition, manuscript development, and manuscript writing; SK: manuscript development, manuscript writing, and manuscript authorization.

## Conflict of Interest Statement

This study is conducted in the absence of any commercial or financial relationships that could be construed as a potential conflict of interest.
